# Sarcopenia Is a Negative Prognostic Factor in Patients Undergoing Transarterial Chemoembolization (TACE) for Hepatic Malignancies

**DOI:** 10.3390/cancers11101503

**Published:** 2019-10-08

**Authors:** Sven H. Loosen, Maximilian Schulze-Hagen, Philipp Bruners, Frank Tacke, Christian Trautwein, Christiane Kuhl, Tom Luedde, Christoph Roderburg

**Affiliations:** 1 Department of Medicine III, University Hospital RWTH Aachen, Pauwelsstrasse 30, 52074 Aachen, Germany; sloosen@ukaachen.de (S.H.L.); ftacke@ukaachen.de (F.T.); ctrautwein@ukaachen.de (C.T.); 2 Department of Diagnostic and Interventional Radiology, University Hospital RWTH Aachen, Pauwelsstraße 30, 52074 Aachen, Germany; mschulze@ukaachen.de (M.S.-H.); pbruners@ukaachen.de (P.B.); ckuhl@ukaachen.de (C.K.); 3 Department of Hepatology and Gastroenterology, Charité University Medicine Berlin, Augustenburger Platz 1, 10117 Berlin, Germany; 4 Division of Gastroenterology, Hepatology and Hepatobiliary Oncology, University Hospital RWTH Aachen, Pauwelsstrasse 30, 52074 Aachen, Germany

**Keywords:** HCC, TACE, cancer, muscle weight, sarcopenia

## Abstract

Background and Aims: While transarterial chemoembolization (TACE) represents a standard of therapy for intermediate-stage hepatocellular carcinoma (HCC) and is also routinely performed in patients with liver metastases, it is still debated which patients represent the ideal candidates for TACE therapy in terms of overall survival. Sarcopenia, the degenerative loss of skeletal muscle mass and strength, has been associated with an adverse outcome for various malignancies, but its role in the context of TACE has largely remained unknown. Here, we evaluated the role of sarcopenia on the outcome of patients undergoing TACE for primary and secondary liver cancer. Methods: The patients’ psoas muscle size was measured on axial computed tomography (CT) scans and normalized for the patients’ height squared. This value was referred to as the psoas muscle index (PMI). The PMI was correlated with clinical and laboratory markers. Results: While pre-interventional sarcopenia had no impact on the direct tumor response to TACE, sarcopenic patients with a pre-interventional PMI below our ideal cut-off value of 13.39 mm/m^2^ had a significantly impaired long-term outcome with a median overall survival of 491 days compared to 1291 days for patients with a high PMI. This finding was confirmed by uni- and multivariate Cox-regression analyses. Moreover, a progressive rapid decline in muscle mass after TACE was a predictor for an unfavorable prognosis. Conclusion: Our data suggest that sarcopenia represents a previously unrecognized prognostic factor for patients undergoing TACE therapy which might yield important information on the patients’ post-interventional outcome and should therefore be implemented into clinical stratification algorithms.

## 1. Introduction

For many patients with primary (e.g., hepatocellular carcinoma) and secondary liver tumors (e.g., metastases of gastrointestinal cancers), surgical tumor resection is not feasible due to advanced tumor stage or limited liver function at the time of tumor diagnosis [1,2]. For these patients, transarterial chemoembolization (TACE) has evolved as a routinely performed treatment modality, providing local tumor control without major systemic side effects [3]. However, response rates to TACE are heterogeneous and the appropriate selection of patients who will benefit particularly well from TACE in terms of overall survival is still debated [4,5]. In the past, different single factors, such as the hepatic functional reserve, tumor distribution and size as well as laboratory parameters have been suggested for patient selection in the context of TACE [4,5]. Moreover, predictive algorithms such as the assessment for retreatment (ART) score [6,7] or SNACOR (tumour Size and Number, baseline Alpha-fetoprotein, Child-Pugh and Objective radiological Response)) score [8], which are mainly based on imaging and on liver function, were suggested for guiding therapeutic decisions in these patients. However, since none of these parameters could be reliably validated as predictive and/or prognostic factors in larger clinical trials, patient allocation to TACE therapy is mainly still based on individual clinicians’ decision in daily routine, corroborating the vital need for novel pre-interventional stratification strategies.

Sarcopenia has been defined as the “progressive loss of muscle mass and strength with a risk of adverse outcomes such as disability, poor quality of life and death” by the Special Interest Group of the European Sarcopenia Working Group in 2010 [9]. In patients with malignant diseases, muscle loss is induced by a systemic inflammatory response leading to decreased appetite, increased catabolism and immobility [10,11]. Muscle loss was recently associated with an impaired prognosis in patients with different solid tumors [12]. In patients with hepatocellular carcinoma (HCC) and colorectal cancer, sarcopenia has been associated with an impaired overall- and disease-free survival after surgical resection or radiofrequency ablation [13,14]. However, the impact of sarcopenia on tumor response and overall survival in patients receiving TACE therapy in a palliative intent has not been sufficiently assessed to date. We therefore aimed at evaluating a potential role of sarcopenia as an easily accessible marker for predicting the clinical outcome of patients undergoing TACE for primary and secondary liver cancer.

## 2. Patients and Methods

### 2.1. Design of the Study and Patient Characteristics

This observational cohort study was conducted to analyze a potential impact of sarcopenia on treatment response as well as overall survival following TACE therapy in patients with primary (HCC) or secondary (metastases) liver cancer. 56 patients who received TACE therapy at the Department of Diagnostic and Interventional Radiology at University Hospital RWTH Aachen between 2013 and 2018 were subsequently enrolled into this study (see Table 1 for patient characteristics). The protocol of the study was approved by the ethics committee of the University Hospital RWTH Aachen (RWTH Aachen University, Germany) under the EK335/17. The study was conducted in accordance with the ethical standards laid down in the Declaration of Helsinki.

### 2.2. Transarterial Chemoembolization (TACE)

Hepatic tumors were treated with an emulsion of a chemotherapeutic agent and an embolic agent diluted with iodized contrast (Ultravist 300, Bayer Vital GmbH, Leverkusen, Germany). For HCCs, a combination of doxorubicin and ethiodized oil (Lipiodol, Guerbet LLC, Bloomington, IN, USA) was used. Liver metastases from colorectal, gastric or pancreatic cancer were treated using a chemotherapeutic agent in accordance with the respective guidelines as well as degradable starch microspheres (EmboCept S, PharmaCept GmbH, Berlin, Germany) or drug eluting beads (DcBeads, BTG International Ltd, London, UK). TACE procedures were conducted via the right femoral artery. A hepatography and a contrast-enhanced cone-beam computed tomography (CT) scan in late arterial contrast phase were performed using a distinct microcatheter. Depending on the tumor type and the number, size, localization and arterial supply of the tumor, a superselective (subsegmental), selective (segmental) or non-selective (lobar) approach was performed.

### 2.3. TACE Response Analysis

TACE response was analyzed on either a multidetector CT scan with multiphasic, contrast-enhanced acquisitions in unenhanced, arterial, portal venous and late-venous phase or a multiphasic, contrast enhanced liver magnetic resonance imaging (MRI) (1.5T, Philips Medical Systems DMC GmbH, Hamburg, Germany). Baseline imaging was conducted not earlier than 28 days before and at about four weeks after the TACE procedure. The median duration between the TACE procedure and the follow-up imaging scan for the assessment of tumor response was 34 days. To evaluate tumor response to TACE, CT and MRI scans were analyzed based on RECIST 1.1 criteria for non-arterially enhanced tumor entities (liver metastases) [15] and mRECIST criteria for HCC [16]. HCC tissue was differentiated from lipiodol deposition by comparison of unenhanced and arterially enhanced contrast scans. Overall tumor response was graded according to the RECIST 1.1/mRECIST standard nomenclature: Complete response (CR), partial response (PR), stable disease (SD) and progressive disease (PD). Complete response and partial response were defined as an objective response (OR) [17].

### 2.4. Assessment of Sarcopenia and Psoas Muscle Index

**Figure 1:**
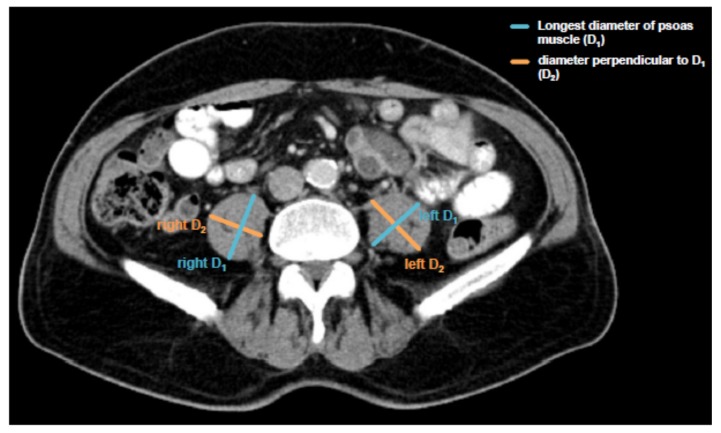
Assessment of the psoas muscle index (PMI). Sarcopenia was assessed by measuring the longest diameter (D_1_) and the perpendicular diameter (D_2_) of the right (ri) and left (le) psoas muscle on an axial computed tomography (CT) scan in the same plane and normalizing it for the patients’ height squared. This value is referred to as the psoas muscle index (PMI).

Sarcopenia was assessed by measuring the longest diameter (D_1_) and the perpendicular diameter (D_2_) of the right (ri) and left (le) psoas muscle on an axial CT scan. All diameters were measured in the same CT plane, which was usually between lumbar vertebral body (LVB) 3 and LVB 4. The individual course of sarcopenia after TACE therapy was assessed accordingly using the same CT plane. The median time to the follow-up scan after TACE therapy for the assessment of sarcopenia was 57.5 days (IQR: 43.75–73 days). An exemplary image of the psoas muscle measurement is displayed in Figure 1. All measurements were then added and divided by four. To normalize the muscle mass for the patient’s height it was divided by the square of the patients’ height. The psoas muscle index (PMI) was consequently defined as following:

$$\mathrm{PMI}\;[\mathrm{mm}/m^{2}]=\frac{(\mathrm{ri}D_{1}\left[\mathrm{mm}\right]+\mathrm{ri}D_{2}\left[\mathrm{mm}\right]+leD_{1}\left[\mathrm{mm}\right]+leD_{2}\left[\mathrm{mm}\right])/4}{\mathrm{patients}’\;\mathrm{height}\;{\left[m\right]}^{2}}$$

### 2.5. Measurement of Cytokine Serum Levels

Serum levels of interleukin (IL)-6 and IL-10 were analyzed using a multiplex immunoassay as described previously [18]. Measurements were performed on a Bio-Plex 200 system using Bio-Plex Manager 6.0 software (Bio-Plex Pro Human Chemokine Panel, #171AK99MR2, Bio Rad, Hercules, CA, USA).

### 2.6. Measurement Laboratory Parameters

Standard laboratory markers were measured in the laboratory center for blood analyses at University Hospital RWTH Aachen. Standard haematological and clinical chemistry parameters were measured using the Sysmex XN9000 (Sysmex GmbH, Norderstedt, Germany) and the Cobas 8000 c701 (Hoffmann-La Roche AG, Basel, Switzerland).

### 2.7. Statistical Analysis

Statistical analyses were performed as recently described [19]. In detail, Shapiro-Wilk test was performed to test for normal distribution of data. Non-parametric data for two groups were compared using the Mann-Whitney-U-Test. Receiver operating characteristic (ROC) curves were created by plotting sensitivity against 1-specificity. The predictive value of the PMI with respect to an objective response to TACE therapy was also evaluated using binary logistic regression. We plotted Kaplan-Meier curves to display the overall survival of patient subgroups with respect to different patient characteristics (e.g., PMI). The optimal cut-off value with respect to overall survival was calculated with a recently published biometric software, which fits Cox proportional hazard models to the survival status and the survival time [20]. The prognostic relevance of individual variables was also tested by univariate and multivariate Cox-regression analyses. Parameters with *p*-value < 0.2 in univariate analysis were included into multivariate analysis. The hazard ratio (HR) and the 95% confidence interval are displayed. Correlation analyses were performed using the Pearson correlation coefficient (r). All statistical analyses were performed with SPSS 23 (SPSS, Chicago, IL, USA) [21]. A *p*-value of <0.05 was considered statistically significant (* *p* < 0.05; ** *p* < 0.01; *** *p* < 0.001).

## 3. Results

### 3.1. Patient Characteristics

A total of 56 patients who underwent TACE therapy for either HCC (*n* = 46) or liver metastases (*n* = 10) were included into this study. 34.8%, 58.7% and 6.5% of HCC patients showed a Barcelona Clinic Liver Cancer (BCLC) A, B or C tumor stage, respectively. The majority of HCC patients (80.4%) revealed an underlying Child-Pugh-A liver cirrhosis, while 17.4% of patients had Child-Pugh-B status and 2.2% had no signs of liver cirrhosis. The median age of the study population was 65 years (range: 30–89 years). 41.5% of patients showed an objective response to TACE therapy (see Patients & Methods for details). 60.0% deceased during the follow-up period. The median overall survival (OS) of all patients was 611 days (20.4 month). Patient characteristics are summarized in Table 1.

### 3.2. Psoas Muscle Index (PMI) and Disease Characteristics

We first determined the grade of pre-interventional sarcopenia by measuring the patients’ psoas muscle size on axial CT scans and normalizing it for the height squared (Figure 1, see Patients & Methods for details). This value is from now on referred to as the psoas muscle index (PMI), a surrogate for sarcopenia. The median PMI in our cohort was 11.81 mm/m^2^ (range: 7.96–16.76 mm/m^2^). 

**Figure 2:**
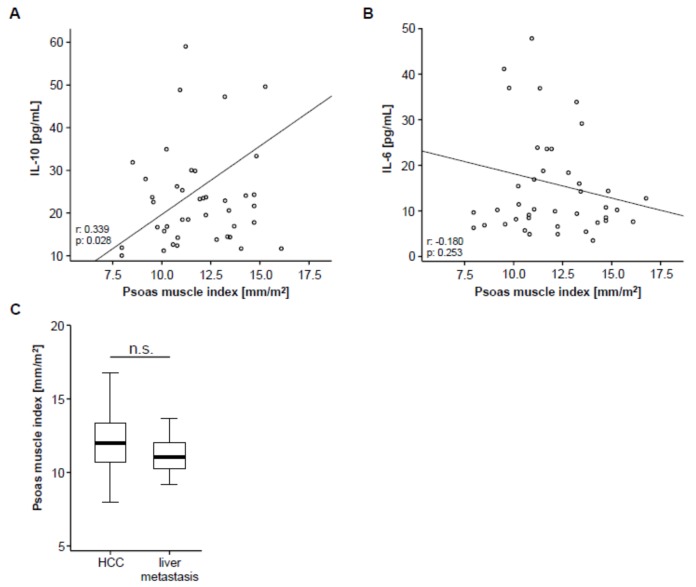
PMI and systemic inflammation. (**A**) The PMI shows a significant correlation with circulating levels of interleukin (IL) IL-10. (**B**) Serum levels of IL-6 show a trend towards a negative correlation with the PMI. (**C**) The PMI is unaltered between patients with primary (hepatocellular carcinoma (HCC), *n* = 46) and secondary liver cancer (liver metastases, *n* = 10).

Based on previous studies arguing for a correlation between systemic inflammation and sarcopenia [22], we next analyzed circulating levels of IL-10 and IL-6 as surrogates for anti- and pro-inflammatory cytokines in our cohort of patients using multiplex immunoassay. Interestingly, we found a significant correlation between the patients’ PMI and circulating levels of IL-10 (Figure 2A), meaning that sarcopenic patients with a low PMI show reduced levels of the anti-inflammatory cytokine IL-10. On the other hand, IL-6 serum levels did not significantly correlate with the PMI but only showed a weak trend towards a negative correlation (Figure 2B), meaning that sarcopenic patients tend to have higher levels of the pro-inflammatory cytokine IL-6.

To further elaborate a potential association between the PMI and other disease characteristics, we subsequently performed correlation analyses including clinical parameters (patients’ age, Charlston Comorbidity Index, size of hepatic target lesion) as well as standard laboratory markers of organ dysfunction (sodium, potassium, leukocytes, platelets, INR, AST, ALT, LDH, GGT, ALP, CRP and albumin). However, in this analysis, we found no significant correlation between these parameters and the patients pre-interventional PMI (Table S1). To exclude a potential influence of the underlying tumor entity on the PMI, we finally compared the PMI between patients with HCC and liver metastasis, which revealed no significant difference between these groups (Figure 2C).

### 3.3. Pre-Interventional Sarcopenia Does Not Correlate with Treatment Response to TACE Therapy

**Figure 3:**
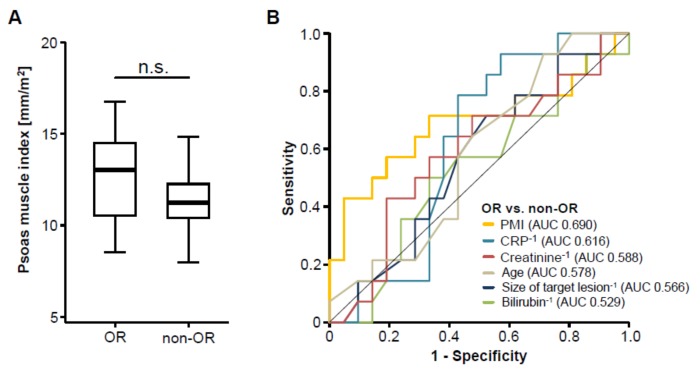
The pre-interventional PMI does not correlate with treatment response to transarterial chemoembolization (TACE) therapy. (**A**) Patients who showed an objective response (OR) to TACE therapy have a similar PMI compared to non-responding (non-OR) patients. (**B**) Receiver operating characteristic (ROC) curve analysis regarding the discrimination between OR and non-OR patients.

We next assessed post-interventional CT scans to evaluate the patients’ tumor response to TACE therapy and subsequently divided the cohort of patients into two groups according to the radiological response, showing either an objective response (OR, complete or partial remission) or not (non-OR, stable or progressive disease), as described previously [17]. To evaluate whether pre-interventional sarcopenia has an impact on the patients’ response to TACE therapy, we next compared the PMI between patients with an OR and non-OR patients. Interestingly, we found no significant difference in the patients’ PMI between these two groups (Figure 3A). In line, ROC curve analysis revealed that the PMI was unsuitable to distinguish between OR and non-OR patients, showing an area under the curve (AUC) value of 0.690 (Figure 3B). However, the PMI was numerically higher to laboratory markers of inflammation (AUC_CRP_: 0.616), liver function (AUC_Bilirubin_: 0.529), kidney function (AUC_Creatinine_: 0.588) as well as clinical parameters such as the pre-interventional size of the target lesion (AUC: 0.566) or the patients’ age (AUC: 0.578, Figure 3B). Finally, we performed univariate binary logistic regression analysis to further investigate the impact on sarcopenia on the treatment response to TACE therapy. Although we observed a trend towards statistical significance, this analysis showed that the pre-interventional PMI was not able to predict an objective response following TACE therapy (Odds ratio: 0.704, 95% CI: 0.494–1.003, *p* = 0.052).

### 3.4. Pre-Interventional Sarcopenia Is Associated with a Reduced Overall Survival after TACE

**Figure 4:**
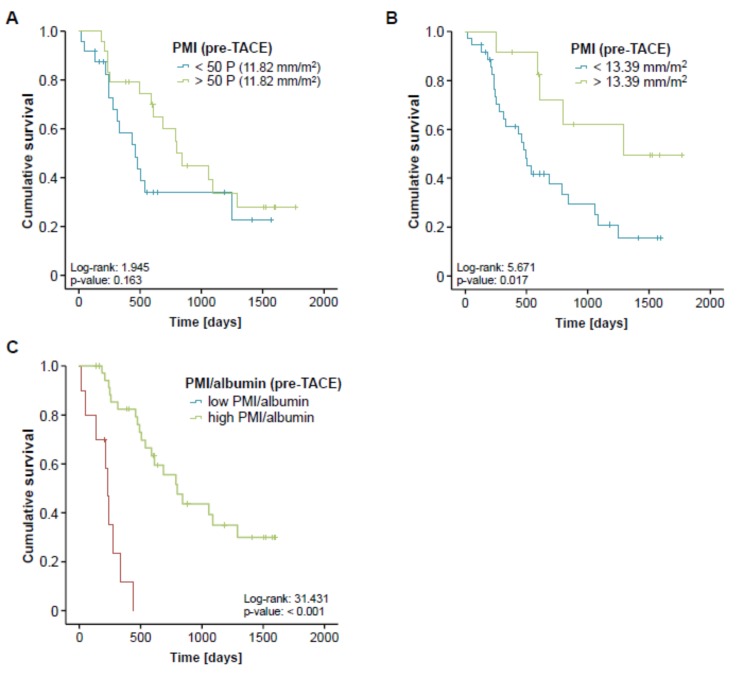
Pre-interventional sarcopenia is associated with a reduced overall survival after TACE. (**A**) Patients with a pre-interventional PMI below the 50th percentile show a trend towards an impaired prognosis. (**B**) Kaplan-Meier curve analysis shows a significantly reduced overall survival for sarcopenic patients with a pre-interventional PMI below 13.39 mm/m^2.^ The median OS in this group was 491 days (16.4 month) compared to 1291 days (43 month) in patients with a PMI above this ideal cut-off value. (**C**) Patients with a low combined PMI*albumin score show a strikingly reduced overall survival of 234 days (7.8 month).

Although sarcopenia seemed to have no impact on the direct treatment response to TACE therapy, we next evaluated whether the PMI might be associated with the patients overall survival (OS). Therefore, we divided our cohort of TACE patients into two subgroups with respect to their pre-interventional PMI (either above or below the 50th percentile). When applying this cut-off value, Kaplan-Meier curve analysis revealed a trend towards a significantly impaired OS in sarcopenic patients with a low PMI (<11.82 mm/m^2^, Figure 4A). The median OS in this group was 460 days (15.3 month) compared to 799 days (26.6 month) in patients with a high PMI (>11.82 mm/m^2^). Taking into account that the 50th percentile might not represent the ideal cut-off value to discriminate between patients with a good or poor prognosis, we next established an optimal prognostic PMI cut-off value by fitting Cox proportional hazard models to the survival time and status and subsequently testing for the PMI value with the most significant difference in the log-rank test as previously described [20], which revealed a PMI value of 13.39 mm/m^2^. When we applied this ideal cut-off value, Kaplan-Meier curve analysis displayed a significantly reduced OS for sarcopenic patients with a pre-interventional PMI below 13.39 mm/m^2^ (Figure 4B). These patients showed a strikingly reduced median OS of just 491 days (16.4 month) compared to 1291 days (43 month) in patients with a PMI above the ideal cut-off value.

In order to further elaborate the prognostic value of sarcopenia in the context of TACE therapy, we next performed univariate Cox-regression analysis with respect to the patients’ OS. Importantly, sarcopenia, again defined by a PMI below our ideal cut-off value, turned out as a negative prognostic factor for OS, showing a hazard ratio (HR) of 3.075 (*p* = 0.023, 95% CI: 1.165–8.117). Finally, we performed extensive univariate Cox-regression analyses on clinicopathological parameters (age, tumor size, sex) as well as laboratory markers of liver (bilirubin, ALT, albumin) and kidney dysfunction (creatinine) or inflammation (CRP, LDH) and included parameters with a p-value of < 0.2 into multivariate analysis (Table 2). Importantly, this analysis revealed that the prognostic impact of a low PMI (<13.39 mm/m^2^) was independent of these parameters (Table 2).

Finally, we hypothesized that the prognostic value of the PMI might even be further increased when combined with other prognostically relevant factors. We therefore combined the PMI with the patients’ pre-interventional albumin serum levels, which also turned out as an independent prognostic factor in multivariate Cox-regression analysis (Table 2), by multiplying both parameters. Again, we established an ideal cut-off value for this prognostic surrogate as described previously. Importantly, Kaplan-Meier curve analysis revealed a strikingly reduced outcome for patients with a low combined PMI*albumin score (low PMI and low albumin levels) showing a median overall survival of 234 days (7.8 month) compared to 799 days (26.6 month) for patients with a high combined PMI*albumin score (Figure 4C). Off note, none of the patients with a low pre-interventional PMI*albumin score reached long-term survival beyond 18 months. In line, the combined PMI*albumin score turned out as a significant predictive factor in Cox-regression analysis (HR 0.939; 95% CI: 0.900–0.981; *p* = 0.004).

### 3.5. Post-Interventional Sarcopenia Is Associated with a Poor Prognosis after TACE

**Figure 5:**
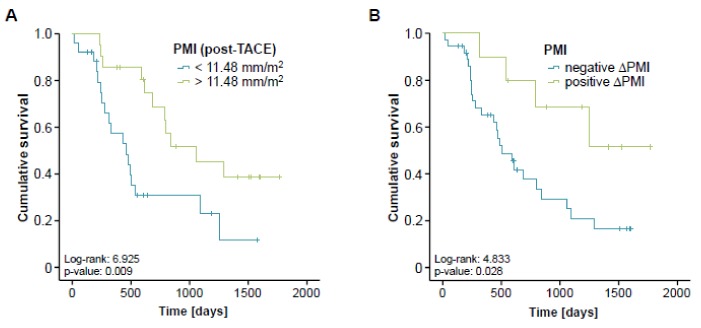
Post-interventional sarcopenia is associated with a poor prognosis after TACE. (**A**) Patients with a post-interventional PMI below the ideal cut-off value (11.48 mm/m^2^) have a significantly impaired long-term survival following TACE therapy. (**B**) Patients with progressive sarcopenia after TACE (negative ∆PMI) show a significantly reduced overall survival compared to patients with a reduced level of sarcopenia after therapy (positive ∆PMI).

Based on the strong association between pre-interventional sarcopenia and a reduced OS, we next analyzed if post-interventional sarcopenia might also predict a poor long-term survival. We therefore assessed the follow-up CT scans after TACE therapy and determined the respective post-interventional PMI for all patients with available follow-up examination (*n* = 47). Again, we established an ideal post-interventional PMI cut-off value as described before. When applying this optimal cut-off value of 11.48 mm/m^2^, Kaplan-Meier curve analysis revealed a significantly impaired long-term survival for patients with a PMI below this cut-off value (Figure 5A). In line, a low post-interventional PMI (<11.48 mm/m^2^) turned out as a negative prognostic factor in univariate Cox-regression analysis showing a HR of 2.722 (*p* = 0.011, 95%CI: 1.275–5.896).

We finally evaluated if longitudinal changes of the PMI over time (between the pre- and post-interventional CT-scan) might yield further information on the patients’ outcome. We therefore divided our cohort of patients into two subgroups either showing an increasing (*n* = 10) or a decreasing (*n* = 37) PMI in the post-interventional CT scan compared to the initial value before TACE therapy. Importantly, we found that patients with progressive sarcopenia after TACE (decreasing PMI, negative ∆PMI) had a significantly reduced overall survival compared to patients with a reduced level of sarcopenia after therapy (increasing PMI, positive ∆PMI, Figure 5B). The median overall survival was 506 days (16.9 month) in the negative ∆PMI group while this endpoint was not reached yet in the positive ∆PMI group. A negative ∆PMI was also a significant negative prognostic factor in univariate Cox-regression analysis revealing a HR of 3.103 (*p* = 0.037, 95% CI: 1.073–8.976).

## 4. Discussion

By using a well characterized cohort of 56 patients undergoing TACE therapy for primary or secondary liver cancer, we demonstrated that the presence of sarcopenia represents a negative prognostic parameter in terms of overall survival for these patients. In contrast, sarcopenia was not predictive for tumor response, highlighting that the influence of sarcopenia on the patients’ outcome did not reflect tumor specific factors but rather the patients’ general clinical condition.

In the last few years, multimodal therapies for primary and secondary hepatic malignancies have considerably evolved [23,24] leading to a significantly improved outcome of patients with liver cancer [24,25]. Beside highly intensive chemotherapies and radical surgical approaches, locally ablative techniques such as transarterial chemoembolization (TACE) have become of increasing importance in the therapy of both primary and secondary liver tumors [26,27,28]. In this context, the question which individual patient represents an optimal candidate for different therapeutic modalities and, in particular, which patient will particularly well benefit, e.g., from TACE therapy, represents a highly debated but still unresolved clinical issue. In general, the individual therapeutic decisions vary significantly between centers, which are not only due to local preferences and experience but also due to a lack of well-established pre-interventional stratification tools. In most centers, the local tumor expansion (e.g., vascular invasion), liver function and clinical scores such as the hepatoma arterial embolization prognostic (HAP) score [29] are considered when deciding whether a patient should receive TACE or not. Here, we show that a low pre-interventional psoas muscle index (PMI), as a surrogate for the presence of sarcopenia which can be calculated in routine pre-interventional staging CT or MRI scans, is indicative for the patients’ post-interventional overall survival (OS). As such, a pre-interventional PMI below our ideal cut-off value of 13.39 mm/m^2^ was an independent predictor for an unfavorable outcome (see Figure 3 and Table 2). Patients with a PMI below this cut-off value showed a median OS of just 491 days (16.4 month) compared to 1291 days (43 month) in patients with a PMI above the ideal cut-off value. Off note, these results are in line with most available studies on an association between sarcopenia and survival in cancer, highlighting the value of the pre-intervention PMI in predicting patients’ prognosis after TACE therapy. Importantly, a combination of the PMI with other prognostically relevant factors such as serum albumin levels, further increased the PMI’s prognostic power arguing that this easily accessible score might also be implemented into existing and future stratification algorithms rather than being used as a stand-alone parameter. Nevertheless, this approach warrants further internal and external validation before a clinical implementation of, e.g., the PMI/albumin score into clinical routine can be considered.

Similar to the association between pre-interventional sarcopenia and a reduced OS, at a cut-off value of 11.48 mm/m^2^, a low post-interventional PMI also turned out as a negative prognostic factor in our cohort of patients. We therefore attempted to answer the clinically more important question whether a longitudinal change of the PMI over time (between the pre- and post-interventional CT-scan) might provide further information on the patients’ outcome. Strikingly, this analysis demonstrated that patients whose PMI was decreasing after TACE had a significantly reduced overall survival compared to patients with an increasing PMI. Importantly, the difference in median OS (negative ∆PMI: 506 days (16.9 month) vs. positive ∆PMI: endpoint not reached yet) was even more apparent than that in patients with a high/low PMI at the pre-interventional time-point, highlighting that a progression of sarcopenia under TACE therapy represents a previously unrecognized prognostic factor in patients receiving TACE. Thus, we suggest that the PMI should not only be integrated into algorithms for the clinical decision making in patients eligible for TACE but might also be used to trigger specific measures in terms of nutritional support in patients with a low PMI. Notably, recent guidelines for the treatment of liver cancer have integrated the use of specific nutritional support strategies in the management of patients with HCC [30]. Exemplary, the supplementation with branched-chain amino acids (BCAAs) and L-carnitine in patients with HCC was suggested to protect from deterioration of liver function and to increase muscle protein synthesis [31,32]. Furthermore, different authors have demonstrated beneficial effects of physical exercise on muscle atrophy and weight loss in patients with HCC [33]. However, further trials will be necessary to demonstrate that these measures are able to preserve skeletal muscle mass in patients receiving TACE and to improve the prognosis of these patients.

Despite the underlying pathophysiological mechanisms linking sarcopenia and especially a negative ∆PMI with an impaired patients’ prognosis is not fully understood, our observation that sarcopenia has no impact on the direct tumor response suggests that other mechanisms are involved in the underlying association between sarcopenia and an impaired patients’ survival. Sarcopenia and especially its end-stage in oncologic patients, the so-called cachexia, are associated with a systemic inflammatory response. In line, we found a significant positive correlation between the pre-interventional PMI and IL-10 serum levels as an example of an anti-inflammatory cytokine and a trend towards a positive correlation between the PMI and IL-6, supporting the association between systemic inflammation and sarcopenia. Therefore, it seems likely that an impaired nutritional state and/or a progressive deterioration of the nutritional state after TACE treatment reflects general adverse conditions (such as a systemic inflammatory response) that may have a considerable impact on patients’ out- comes upon intra-arterial treatments.

Although the present data suggested that PMI might be used to predict patients’ post-interventional outcome, they do not allow answering the question whether an individual patient with a low PMI might have benefitted similarly or even more in terms of long-term survival from a different treatment modality such as systemic treatment or other locally ablative therapies. Moreover, the number of patients included into this study is rather small as this study was designed as an exploratory analysis and therefore needs further validation. Finally, we included both patients with HCC and secondary liver cancer in our analysis. Although this aspect reduces the entity-specific validity of our findings and limits pathophysiological conclusion, it further suggests that the prognostic value of the PMI on patients’ outcome is rather treatment- than entity-related. To gain further insight into a potential entity-specific validity of the PMI, we conclusively performed all analyses in the subgroup of HCC patients alone (*n* = 46, Figure S1). These analyses revealed comparable results with respect to a prognostic relevance of the PMI and especially the PMI/albumin score following TACE therapy in HCC patients only, although statistical significance was not reached for the PMI only (most likely due to the reduced patient number). Again, a further post-interventional decrease of the PMI (negative ∆PMI) was a strong predictor of overall survival. To exclude a potential confounding effect of the patients’ liver function or alpha-fetoprotein (AFP) serum levels in the subgroup of HCC patients, we performed Cox-regression analysis which revealed no impact of bilirubin (HR: 0.634 (0.237–1.679), *p* = 0.365) or AFP (HR: 1.000 (0.999–1.001), *p* = 0.412) serum levels on patients’ overall survival in our cohort. Together, larger confirmatory prospective clinical studies including patients with different malignancies as well as alternative treatment modalities are warranted to exclude further potential confounders in multivariate Cox-regression analysis and to gain further information on a potential treatment predictive value of the PMI in patients with liver cancer, which we hope to have stimulated with this analysis.

## 5. Conclusions

For many patients with HCC or liver metastasized diseases, TACE has evolved as an effective and safe procedure providing local tumor control and improving overall survival. However, response rates to TACE are heterogeneous and no marker for the identification of patients who will benefit particularly well from TACE have been established to date. Here, we establish sarcopenia as a novel parameter identifying a subgroup of patients with an unfavorable prognosis after TACE and propose a score consisting of the psoas muscle index (PMI) and serum albumin concentration for an easy identification of patients that might not benefit from TACE in terms of overall survival. While these data need to be confirmed in further longitudinal clinical trials using independent cohorts, our results might open the door for a potential clinical use of PMI for an optimized selection of potential TACE patients.

## Figures and Tables

**Table 1:** Characteristics of study population. TACE: Transarterial chemoembolization, HCC: Hepatocellular carcinoma, CRC: Colorectal carcinoma, CCA: Cholangiocarcinoma, OR: objective response.

Characteristic	Study Cohort
TACE patients	56
Sex (%):	
male-female	78.6–21.4
Age (years, median and range)	65 (30–89)
Hepatic malignancy (%):	
HCC	82.1
Liver metastasis (CRC)	10.7
Liver metastasis (gastric cancer)	1.8
Liver metastasis (pancreatic cancer)	3.6
Liver metastasis (CCA)	1.8
Size of target lesion (mm, median and range)	27 (10–129)
HCC tumor stage (%):	
BCLC A	34.8
BCLC B	58.7
BCLC C	6.5
Child-Pugh Score (HCC only) (%):	
No liver cirrhosis	2.2
CHILD A:	80.4
5 points	56.5
6 points	23.9
CHILD B:	17.4
7 points	13
8 points	4.4
OR to TACE therapy (%):	
Yes–No	41.5–58.5
Deceased during follow-up (%):	
Yes–No	60.0–40.0
Median overall survival (days/month)	611/20.4

**Table 2:** Univariate and multivariate Cox-regression analysis for the prediction of overall survival. PMI: psoas muscle index, ALT: Alanine transaminase, LDH: Lactate dehydrogenase, CRP: C-reactive protein.

Characteristic	Univariate Cox Regression		Multivariate Cox Regression	
Parameter	*p*-Value	Hazard-Ratio (95% CI)	*p*-Value	Hazard-Ratio (95% CI)
PMI < 13.39 mm/m^2^	0.023	3.075 (1.165–8.117)	0.041	2.876 (1.044–7.922)
Size of target lesion	0.319	1.006 (0.995–1.016)		
Age	0.162	1.021 (0.992–1.052)	0.435	1.017 (0.975–1.060)
Sex	0.878	0.973 (0.405–2.165)		
Creatinine	0.164	1.491 (0.850–2.615)	0.223	1.436 (0.802–2.570)
ALT	0.614	0.998 (0.992–1.005)		
LDH	0.934	1.000 (0.997–1.002)		
Albumin	0.063	0.495 (0.236–1.039)	0.035	0.314 (0.107–0.924)
CRP	0.002	1.026 (1.009–1.042)	0.058	1.019 (0.999–1.039)
Bilirubin	0.696	0.857 (0.395–1.859)		

## Supplementary Materials

The following are available online at https://www.mdpi.com/2072-6694/11/10/1503/s1, Figure S1. Evaluation of the PMI in HCC patients only (*n* = 46). (**A**) Patients who showed an objective response (OR) to TACE therapy have a similar PMI compared to non-responding (non-OR) patients. (**B**) HCC patients with a pre-interventional PMI below the 50th percentile show a trend towards an impaired prognosis. (**C**) Kaplan-Meier curve analysis shows a strong trend towards a reduced overall survival for sarcopenic patients with a pre-interventional PMI below 13.39 mm/m^2^. (**D**) HCC patients with a low combined PMI*albumin score show a strikingly reduced overall survival. (**E**) Patients with a post-interventional PMI below the ideal cut-off value (11.48 mm/m^2^) show a strong trend towards an impaired long-term survival following TACE therapy. (**F**) HCC patients with progressive sarcopenia after TACE (negative ∆PMI) show a significantly reduced overall survival compared to patients with a reduced level of sarcopenia after therapy (positive ∆PMI). Table S1. Correlation analysis between the PMI and variant laboratory markers.

## Abbreviations

TACE	Transarterial chemoembolization
HCC	Hepatocellular carcinoma
CT	Computed Tomography
PMI	Psoas muscle index
RWTH	Rheinisch Westfälische Technische Hochschule
LVB	Lumbar vertebral body
IL-6	Interleukin-6
IL-10	Interleukin-10
ROC	Receiver Operating Characteristic
INR	International Normalized Ratio
AST	Aspartate Transaminase
ALT	Alanine Transaminase
LDH	Lactate dehydrogenase
GGT	Gamma-glutamyl Transferase
ALP	Alkaline phosphatase
CRP	C-reactive protein
ORr	Overall response rate
AUC	Area under the curve
OS	Overall survival
HR	Hazard ratio
MRI	Magnetic resonance imaging
