# Supplementary material for: Sarcopenia Is a Negative Prognostic Factor in Patients Undergoing Transarterial Chemoembolization (TACE) for Hepatic Malignancies
Source: Cancers (Basel). 2019 Oct 8;11(10):1503. doi: 10.3390/cancers11101503 (PMC6827165; doi:10.3390/cancers11101503)
Supplement: Supplementary file 1 [file cancers-11-01503-s001.pdf]

Supplementary Materials: Sarcopenia Is a Negative Prognostic Factor in Patients Undergoing Transarterial Chemoembolization (TACE) for Hepatic Malignancies

Sven H. Loosen, Maximilian Schulze-Hagen, Philipp Bruners, Frank Tacke, Christian Trautwein, Christiane Kuhl, Tom Luedde and Christoph Roderburg

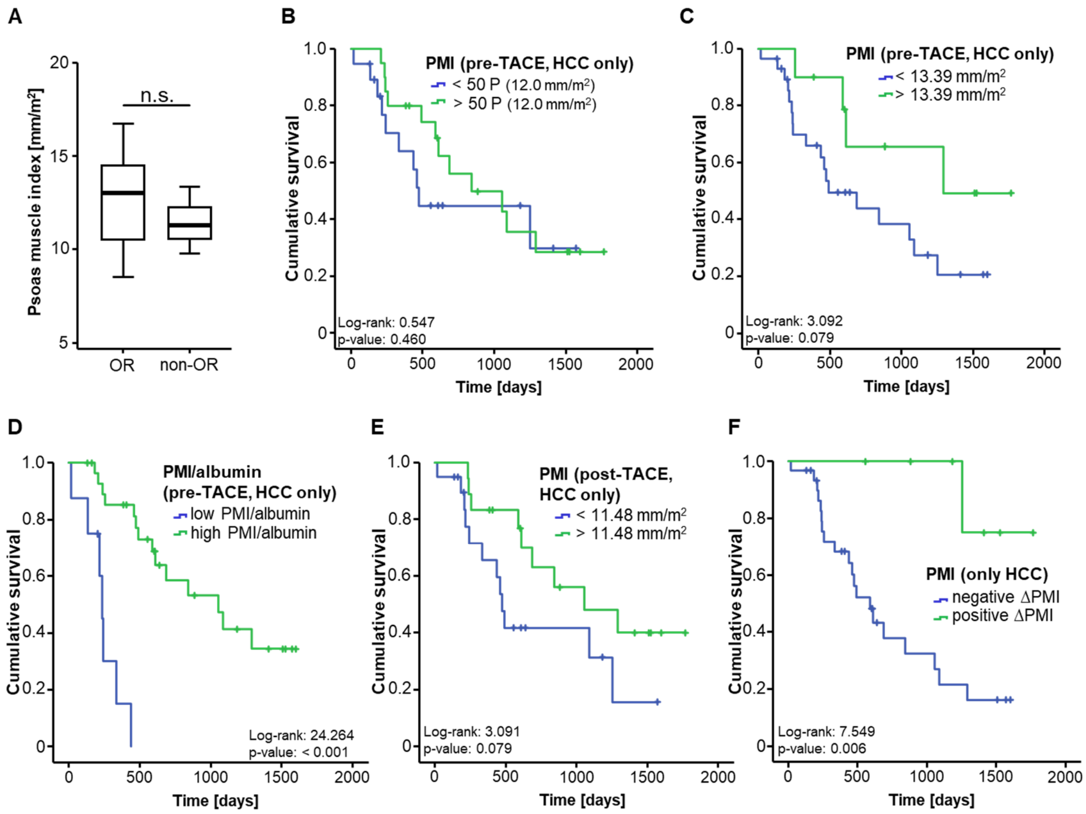

Figure S1. Evaluation of the PMI in HCC patients only ($n = 46$). (**A**) Patients who showed an objective response (OR) to TACE therapy have a similar PMI compared to non-responding (non-OR) patients. (**B**) HCC patients with a pre-interventional PMI below the 50th percentile show a trend towards an impaired prognosis. (**C**) Kaplan-Meier curve analysis shows a strong trend towards a reduced overall survival for sarcopenic patients with a pre-interventional PMI below 13.39 mm/m². (**D**) HCC patients with a low combined PMI*albumin score show a strikingly reduced overall survival. (**E**) Patients with a post-interventional PMI below the ideal cut-off value (11.48 mm/m²) show a strong trend towards an impaired long-term survival following TACE therapy. (**F**) HCC patients with progressive sarcopenia after TACE (negative ΔPMI) show a significantly reduced overall survival compared to patients with a reduced level of sarcopenia after therapy (positive ΔPMI).

Table S1. Correlation analysis between the PMI and variant laboratory markers.

Parameter	Psoas Muscle Index (PMI)	
	r	*p*-value
Age	0.122	0.390
Size of target lesion	0.211	0.174
Charlson Comorbidity Index	−0.040	0.815
Sodium	0.193	0.190
Potassium	−0.039	0.791
Leukocytes	−0.035	0.808
Thrombocytes	−0.075	0.604
INR	0.201	0.167

AST	0.009	0.963
ALT	0.132	0.404
LDH	0.225	0.174
GGT	−0.008	0.956
ALP	−0.081	0.584
CRP	−0.101	0.501
Albumin	0.196	0.182

INR: International Normalized Ratio, AST: aspartate transaminase, ALT: alanine transaminase, LDH: lactate dehydrogenase, GGT: γ-Glutamyl transpeptidase, ALP: alkaline phosphatase, CRP: C-reactive protein, r: Pearson correlation coefficient.
